# Herbst’s Triad as a Manifestation of Gastroesophageal Reflux Disease

**DOI:** 10.1097/PG9.0000000000000066

**Published:** 2021-03-30

**Authors:** Encarnación Torcuato Rubio, Rafael Martín-Masot, Pilar Ortiz Pérez, María Juliana Serrano Nieto, Juan Díaz Ruiz, Javier Blasco-Alonso, Víctor Manuel Navas-López

**Affiliations:** From the * Pediatric Gastroenterology and Nutrition Unit, Hospital Regional Universitario de Málaga, Málaga; † Pediatric Department. Hospital San Pedro de Alcántara, Cáceres; ‡ Primary Care Center, Cártama, Málaga

## BACKGROUND

The Herbst triad (HT) was described in 1976 by Herbst et al. (1) when detecting the association of iron deficiency anemia, hypoalbuminemia and clubbing in 3 children with hiatal hernia and severe esophagitis secondary to gastroesophageal reflux (GER). Although no chronic lung disease associated with reflux was demonstrated to justify the appearance of clubbing, these also disappeared after surgery (1). We present 2 children with HT that required surgical treatment due to lack of response to proton pump inhibitors.

## CLINICAL CASES

**FIGURE 1.**
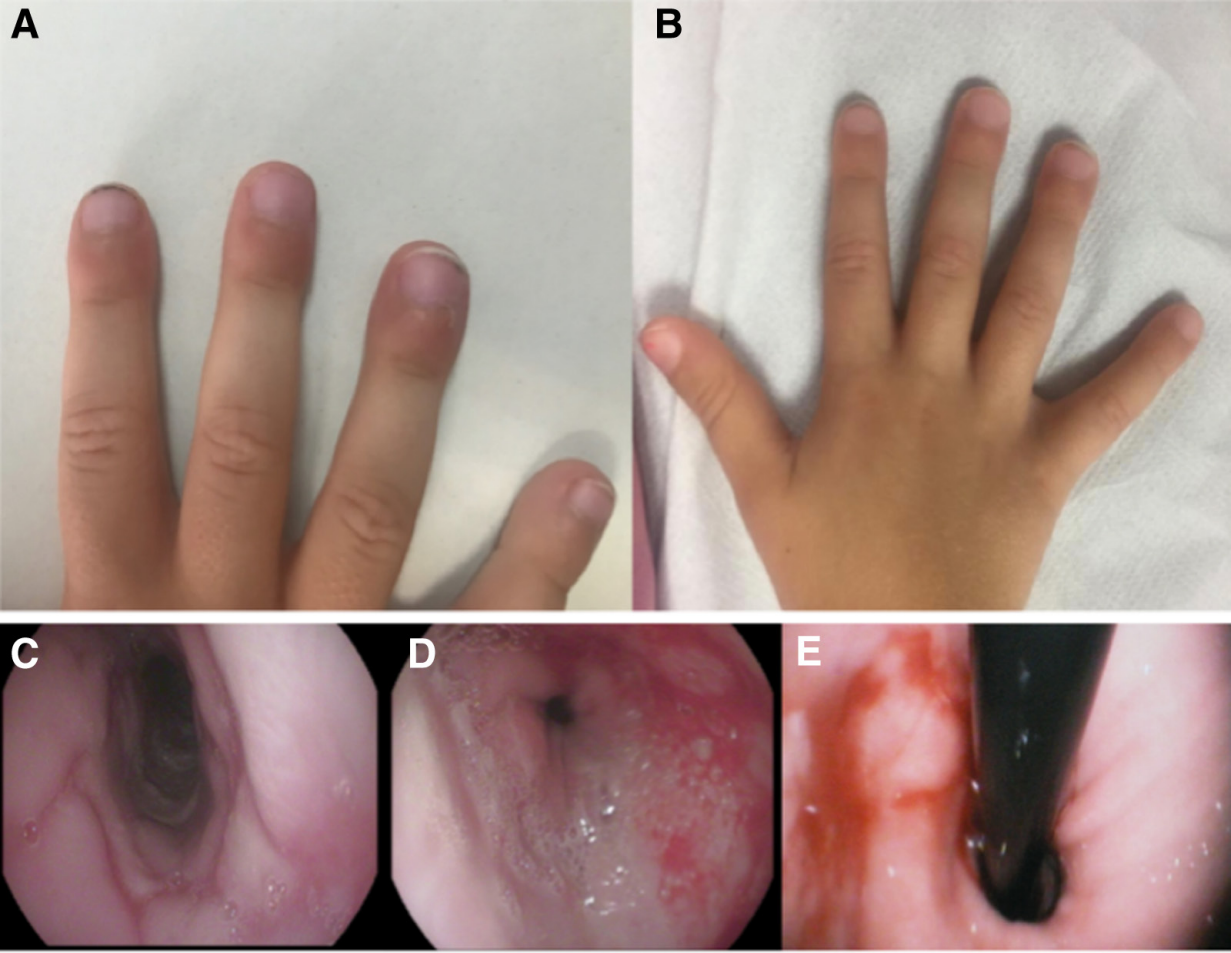
Clinical and endoscopic findings. A) Clubbing at diagnosis. B) Improvement of clubbing at 8 months. C) Middle third of the esophagus with cobblestone appearance. D) Distal third of the esophagus with purulent exudate. E) Sliding hiatal hernia.

The first case corresponds to a 4-year-old girl referred for recurrent brown vomits of one year of evolution, unrelated to the intake. Associated problems were epigastralgia, recurrent episodes of cervical hyperextension, low weight gain, and modification of the eating pattern, eating more frequent but less numerous meals. Upon examination, she had a weight of 14.6 kg (P7, −1.5 SD) and a height of 108 cm (P42, −0.2 SD), scarce adipose panniculus, skin paleness, and clubbing (Fig. 1A). Blood tests highlighted iron deficiency anemia and hypoalbuminemia with normal levels of fecal alpha-1-antitrypsin (Table 1). Esophagogastroduodenoscopy (EGD) showed a decrease in esophageal caliber and wall edema, longitudinal furrows, and discrete trachealization of the upper third of the esophagus, a cobblestone appearance in the middle third (Fig. 1C), and friable mucosa in the lower third with purulent exudate (Fig. 1D), also showing incompetence of the cardiac without hiatal hernia. All of these findings were consistent with grade B esophagitis according to the Los Angeles classification ^(2)^. Histology revealed basal hyperplasia, fibrinopurulent material, and granulation tissue without eosinophils. The histology of stomach and duodenum were normal. Treatment with omeprazole (2 mg/kg/day), oral iron (5 mg/kg/day), and domperidone (0.75 mg/kg/day) was started. After 12 weeks of treatment, symptoms had remitted and progressive improvement of clubbing (Fig. 1B) was observed. Blood test is shown in Table 1. However, 1 year after diagnosis, despite clinical improvement, she could not be weaned from proton pump inhibitor treatment due to recurrence of symptoms. Endoscopy at 12 months showed evidence of peptic esophagitis, a reason why she has been considered a candidate for surgical treatment.

**TABLE 1.** Analytical Values at Diagnosis and After Treatment

	Case 1		Case 2		
	At Diagnosis	After 12 Wks of Treatment	At Diagnosis	After 12 Wks of Treatment	After Surgical Treatment
Hemoglobin (Normal 12–16 g/dL)	7.7	11.3	6.3	8.7	12.5
Platelets (Normal 140 000–450 000/L)	820 000	405 000	329 000	583 000	310 000
Reticulocytes (Normal 25 000–75 000/L)	180 000	97 000	220 000	157 000	66 000
Iron (Normal 40–100 μg/dL)	17	57	9	27	64
Ferritin (Normal 7–140 ng/mL)	1.5	25.3	3	6.7	21
Albumin (Normal 3.8–5.4 g/dL)	1.8	3.1	1.8	2	4
C-reactive protein (Normal < 2.9 mg/L)	2.9	2.9	30.9	24.6	2.9
Antitissue transglutaminase antibodies IgA (Normal < 7 U/mL)	0.30	Not done	1.1	Not done	Not done
Alanine aminotransferase (Normal 7–40 U/L)	31	Not done	20	Not done	Not done
Creatinine (0.20–0.70 mg/dL)	0.28	Not done	0.32	Not done	Not done
Alpha-1-Antitrypsin (Normal < 0.9 mg/g feces)	0.32	—	0.08	—	—
Fecal elastase (Normal > 200 μg/g feces)	—	—	320	—	—
Fecal calprotectin (Normal < 60 μg/g feces)	—	—	108	—	—
Urinalysis	Normal	Not done	Normal	Not done	Not done

The second case corresponds to a child of 2 years and 6 months, with a history of frequent regurgitations in the infant stage, referred for recurrent episodes of periumbilical pain of 15 months of evolution, almost daily and occasional vomiting with food content. In recent months, he was associated with intermittent eyelid edema, progressive asthenia and hyporexia. Physical examination revealed weight 11 kg (P2, −2.01 SD) and height 86.5 cm (P1, −2.26 SD), skin-mucosa pallor, palpebral edema, mild abdominal distention, and clubbing. The blood test showed iron deficiency anemia and hypoalbuminemia with normal levels of fecal alpha-1-antitrypsin (Table 1). Esophageal and gastroduodenal transit was performed, showing hiatal hernia. When the EGD was performed, in addition to the hernia, findings consistent with Los Angeles grade C esophagitis (2) were evidenced (Fig. 1E). Histology revealed intense chronic inflammation, eosinophil infiltration (1–4 eos/hpf), and foci of ulceration with areas of granulation. Treatment with omeprazole (2 mg/kg/day) and sucralfate (3 mg/kg/day) was started, but after 12 weeks, no clinical improvement was observed, persisting low levels of hemoglobin and albumin (Table 1). In the absence of response, a *Nissen* fundoplication was performed 16 weeks after diagnosis, presenting rapid clinical improvement, with normalization of laboratory parameters and disappearance of clubbing (Table 1). At 12 months of evolution, he was asymptomatic.

## DISCUSSION

The clinical manifestations of GER are diverse, epigastralgia, heartburn, and regurgitation (3) in older children and adults, and rumination or Sandifer syndrome in infants and young children (4). To this broad spectrum, HT must be added as an unusual manifestation of GERD (5).

Regarding the pathophysiological mechanism underlying this entity, the erosion of the esophageal mucosa justifies the digestive loss of hemoglobin and albumin (1). Furthermore, plasma protein normalization after specific treatment corroborates the association between reflux esophagitis and protein-losing enteropathy (6). Despite this situation of protein-losing enteropathy, the values of fecal alpha-1-antitrypsin remains in normal range due to 2 mechanisms, intermittent losses of this protein (7) and its degradation in the acidic environment of the stomach.

The underlying mechanism of clubbing is not well defined despite its association with multiple entities and having been studied on numerous occasions (8). Three pathophysiological causes related to vascular endothelial growth factor (VEGF) are postulated: distal phalangeal hypervascularity, since platelet precursors are not fragmented in the pulmonary circulation by venoarterial shunts and are trapped in the distal circulation causing release of VEGF (9); chronic hypoxia, when there is increased expression of VEGF (10); and chronic states of inflammation, in which increased prostaglandins activate endothelial cells and VEGF (11) transcription. These arguments would explain that the cases of GER associated with microaspirations have chronic damage to the respiratory epithelium and a sustained state of inflammation that justifies the appearance of clubbing. However, in none of the described cases of HT described, chronic lung disease, hypoxemia, or venoarterial shunts that justify its presence have been found (1,6,7,12), so its pathophysiological mechanism remains enigmatic. However, due to the uniqueness of the disappearance of clubbing when treating GERD, it is reasonable to attribute its appearance to the presence of esophagitis (5,12).

After reviewing the literature, we found 14 cases of HT published, all in children under 14 years of age, the majority under 7 (1,5–7,12–18) which suggests that this condition mainly affects children (Table 2). This could be justified by the low suspicion of GER in early childhood, given that the classic clinical picture is infrequent at these ages, causing diagnostic delay and perpetuating the pathophysiological mechanism. After diagnosis, in most cases, treatment with prokinetics, omeprazole, and/or H2 antagonists was started for a minimum of 12 weeks. Response to medical treatment was only described in 1 patient after therapy with omeprazole and domperidone for 9 months (15). However, the majority required surgical intervention.

**TABLE 2.** Described Cases of Herbst Triad

Author, Year	Sex	Age (y)	Time From Symptoms Onset to Diagnosis	Hiatal Hernia	Medical Treatment	Duration Medical Treatment (mo)	Type of Surgery	Outcome
Herbst et al 1976 (1)	Boy	4.5	18 mo	Yes	Yes	Unknown	Nissen-Boerema	Healing
	Boy	5.5	From birth	Yes	No	—	Unknown	Healing
	Boy	11	From infancy	Yes	Yes	Unknown	Unknown	Healing
Sacher et al 1990 (6)	Girl	4	3	No	Yes (H2 antagonists)	Unknown	Nissen fundoplication	Healing
	Girl	1.5	Unknown	No	Yes (H2 antagonists and prokinetics)	4	Nissen fundoplication	Healing
Spoulou et al 1995 (7)	Girl	7	14 mo	No	Yes (prokinetics)	6	Nissen fundoplication	Healing
Greenwald et al 1996 (18)	Girl	9	3 y	Yes	Yes (PPIs and H2 antagonists)	6	Nissen fundoplication	Healing
Rosário et al 1998 (17)	Boy	4	1 y	No	Unknown	Unknown	Unknown	Unknown
Calçado et al 2004 (15)	Boy	6	11 mo	Yes	Yes (PPIs and prokinetic)	9	Awaiting surgery at time of publication	Unknown
Guerrero Vázquez et al 2007 (5)	Girl	6	5	Yes	Yes	Unknown	Nissen fundoplication	Healing
Mattiuzzo et al 2007 (14)	Boy	8	Unknown	No	Yes (PPIs, prokinetic and H2 antagonists)	3	Toupet fundoplication	Healing
Piras et al 2010 (12)	Boy	6	Unknown	No	Unknown	Unknown	Nissen fundoplication	Healing
Sachin et al 2019 (13)	Boy	13	1 mo	Yes	Yes	—	Not specified	Healing
Plata-Garcia et al 2020 (16)	Girl	9	5 y	Yes	No	—	Nissen fundoplication	Healing

PPI = proton pump inhibitor.

To conclude, a high suspicion of this disease is important to make an accurate and early diagnosis due to the consequences derived from therapeutic delay, which can cause complications such as esophageal stenosis and Barret’s esophagus (1,7). It is important to highlight the existence of GER in children under 8 years of age in the absence of typical symptoms, as well as the need to include esophagitis secondary to GERD in the differential diagnosis of clubbing.

Parents of the children in question aware of this Case Report and have they given their consent.
